# Research on Absolute Calibration of GNSS Receiver Delay through Clock-Steering Characterization

**DOI:** 10.3390/s20216063

**Published:** 2020-10-25

**Authors:** Feng Zhu, Huijun Zhang, Luxi Huang, Xiaohui Li, Ping Feng

**Affiliations:** 1 National Time Service Center, Chinese Academy of Science, Xi’an 710600, China; zhj@ntsc.ac.cn (H.Z.); huangluxi@ntsc.ac.cn (L.H.); xiaohui@ntsc.ac.cn (X.L.); pingfp@ntsc.ac.cn (P.F.); 2 Technology and Engineering Center for Space Utilization, University of Chinese Academy of Science, Beijing 100039, China; 3 Key Laboratory of Precise Navigation and Timing Technology, Chinese Academy Science, Xi’an 710600, China

**Keywords:** receiver delay, absolute calibration, clock-steering, pulse-per-second (PPS), TtC (time-to-code), TtP (time-to-phase)

## Abstract

The receiver delay has a significant impact on global navigation satellite system (GNSS) time measurement. This article comprehensively analyzes the difficulty, composition, principle, and calculation of GNSS receiver delay. A universal method, based on clock-steering characterization, is proposed to absolutely calibrate all types of receivers. We use a hardware simulator to design several experiments to test the performance of GNSS receiver delay for different receiver types, radio frequency (RF) signals, operation status and time-to-phase (TtP). At first, through the receivers of Novatel and Septentrio, the channel delay of Septentrio is 2 ns far lower than 65 ns for Novatel, and for the inter-frequency bias of GLONASS L1, Septentrio tends to increase within 10 ns compared with decreasing of Novatel within 5 ns. Secondly, a representative receiver of UniNav-BDS (BeiDou) is chosen to test the influence of Ttp which may be ignored by users. Under continuous operation, the receiver delay shows a monotone reduction of 10 ns as TtP increased by 10 ns. However, under on-off operation, the receiver delay represents periodic variation. Through a zero-baseline comparison, we verifies the relation between receiver delay and TtP. At last, the article analyzes instrument errors and measurement errors in the experiment, and the combined uncertainty of absolute calibration is calculated with 1.36 ns.

## 1. Introduction

The calibration of global navigation satellite systems (GNSS) receiver delay is always a difficult field in navigation [1]. Due to different characterizations of receivers, there are also differences in calibration methods, which should be studied with pertinence solutions [2,3]. At present the most widely used method is the relative calibration developed by Bureau International des Poids et Mesures (BIPM), with the advantages of simple operation and high precision, which is mainly used in timing transfer field such as GNSS common-view (CV) or two-way satellite time and frequency transfer (TWSTFT) [4,5]. However, this method can only obtain the relative delay between the calibrated receiver and the reference receiver [6]. In some applications such as one-way timing, timing evaluation, and time-offset monitoring, the receiver delay must be accurately obtained through absolute calibration [7,8].

The absolute calibration method is first proposed by the Colorado university and put into operation by Naval Research Laboratory (NRL), which can absolutely calibrate GNSS receiver delay for different frequency band, code type, and signal modulation by using a GNSS hardware simulator [9,10,11]. In most experiments the receivers selected by NRL represented the external 10 MHz and pulse-per-second (PPS) from the atomic clock. As the reference time fixed the receiver delay stably, the variation of calibration result is mostly focused on temperature sensitivity, reference clock, source bandwidth, and data processing [12,13,14]. Nevertheless, the phase difference between external 10 MHz and PPS named time-to-phase (TtP) also affected the calibration result [15]. Although several related papers from NRL or Centre National d’Etudes Spatiales (CNES) had analyzed the relation between receiver delay and TtP, there is still lack of comprehensive research on its principle and method [9,16]. Meanwhile, especially for the timing receivers without PPS input, hardly any papers had analyzed the calibration method, which should also be studied as key research.

In order to research the absolute calibration method of GNSS receivers delay, the article first analyzed the timing characterization of GNSS receiver delay. Through dividing the receiver delay into parts, we presented the method of absolute calibration based on the clock-steering characterization. Using a GNSS hardware simulator of Spirent GSS8000 to establish an absolute calibration platform. Two types of GNSS receivers like Novatel and Septentrio, both without PPS input, are calibrated for comparison. After self-calibration of the simulator, the experiment absolutely calibrated the channel delay of GPS L1/L2 and inter-frequency bias of GLONASS L1. Through comparing the calibration result of PPS output delay, we specially tested the clock-steering performance of receiver PPS output. As a representative receiver with external 10 MHz and PPS, UniNav-BDS (BeiDou) is absolutely calibrated with the variation of TtP. A zero-baseline comparison verified the relation between calibration result and TtP under the case of continuous operation and on–off operation. Finally, we analyzed the calibration uncertainty, and the discussion is given.

## 2. Timing Characterization of GNSS Receiver Delay

As time equipment, each GNSS receiver must have a receiver time which is short for RecT, and especially for well-timing receivers, RecT has not only high stability but also keeps synchronous to a unified time scale [17]. After successful positioning, the receiver always represents RecT with PPS output for user timing, and most measurement values from GNSS receiver are generated under RecT drive. In order to comprehend the composition of receiver delay better, we analyze the working process of RecT.

### 2.1. Clock-Steering Characterization

**Figure 1:**
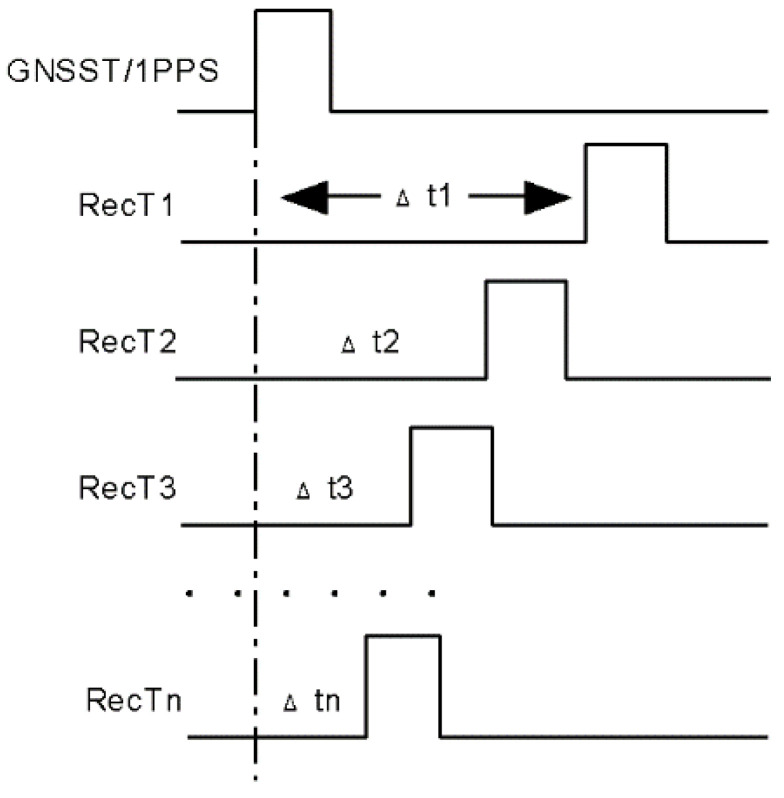
Clock-steering processing of the receiver time.

RecT has been generated by the receiver clock such as an oscillator since the receiver starts operating. As the rising edge of RecT is random, the receiver will reduce the clock-bias by adjusting RecT to GNSS time (GNSST) or PPS input, which is named clock-steering processing as shown in Figure 1 [18].

At the beginning of receiver operation, RecT is frequently divided into generation by the receiver clock at any time. In order to synchronize RecT with a stable time, the receiver will adjust RecT1 by clock-bias $\Delta t_{1}$ after first positioning. If the receiver does not have PPS input, RecT will keep synchronous to global navigation satellite systems time (GNSST) such as GPS time (GPST), otherwise PPS input instead. Ideally $\Delta t_{n}$ is equal to zero as RecT tends to be stable. However, due to hardware configuration $\Delta t_{n}$ can only be accurate within a certain range, such as receiver clock of 100 MHz only accurate to 10 ns, which exists an unknown delay in pseudorange. For some receivers such as Septentrio $\Delta t_{n}$ has already been deducted from the original measurement, which means the pseudorange is a modified value via clock-bias correction.

### 2.2. Composition of the Receiver Delay

The receiver delay commonly refers to the time bias from radio frequency (RF) signal input to measurement data output. For the timing receiver, there is also PPS output which always synchronizes with the measurement data, in other words, the timing receiver delay can be defined as the time bias from RF signal input to PPS output [10,19].

**Figure 2:**
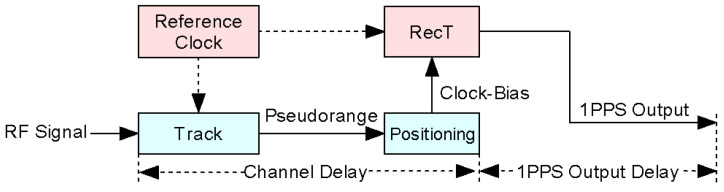
Composition of the receiver delay.

Altogether, GNSS receiver uses the clock-bias to adjust RecT after until clock-bias under the minimum range, which represents RecT with PPS output. Therefore, we divide GNSS receiver delay into two parts as shown in Figure 2. One is the channel delay existing in pseudorange, the other is PPS output delay caused by hardware link. Compared with the relatively fixed PPS output delay, the channel delay varies from different RF signals, reference clocks, parameter settings, etc.

## 3. Method of Absolute Calibration on GNSS Receiver Delay

**Figure 3:**
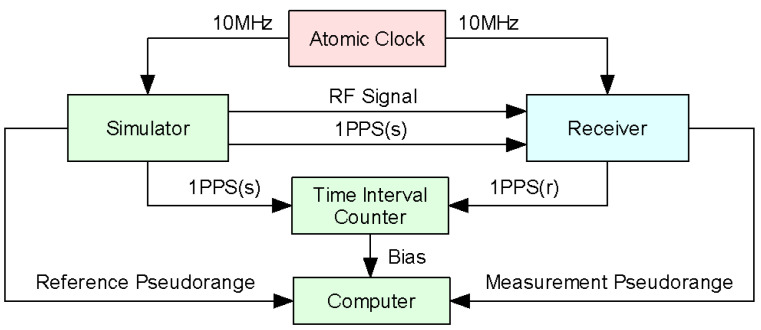
Principle of absolute calibration.

Using a GNSS hardware simulator instead of the real satellites, the principle of absolute calibration is shown in Figure 3. An atomic clock provides the simulator and receiver with the reference clock, and through sending PPS and RF signal to GNSS receiver, the simulator should be self-calibrated at first. A time interval counter measures the bias of PPS output between the receiver PPS(r) and simulator PPS(s). Therefore the data is collected by the computer.

In order to analyze the principle of absolute calibration better, we show the calculation process of the receiver delay as follows.

1. The reference pseudorange represents the ideal distance from the satellite to the receiver, which can be expressed by the formula as below:
  (1) $$\rho_{s}=R+c\cdot \left(\tau_{clk}+\tau_{iono}+\tau_{trop}\right)$$
  where $\rho_{s}$ is the reference pseudorange from the simulator, *R* is the geometry path from the satellite to the receiver, $\tau_{clk}$ is the satellite clock-bias, $\tau_{iono}$ and $\tau_{trop}$ are relatively the ionosphere delay and troposphere delay.

$\rho_{s}$ is calculated by the simulator based on its system time like PPS(s), but it exists the simulator delay between RF signal and PPS(s), which is mainly caused by simulator channel and transmission cable.

2. The actual pseudorange reaching receiver includes the reference pseudorange and simulator delay:
  (2) $$\rho_{s}{}^{\prime }=\rho_{s}+c\cdot \tau_{sim}=R+c\cdot \left(\tau_{clk}+\tau_{trop}+\tau_{iiono}+\tau_{sim}\right)$$
  where $\rho_{s}{}^{\prime }$ is the actual reference pseudorange, $\tau_{sim}$ is the simulator delay between zero-value of RF signal and PPS(s) named time-to-code (TtC), which can be measured from the code phase reversal point relative to rising edge of PPS by an oscilloscope [20].
3. The measurement pseudorange represents the actual distance of signal transmission process, which can be expressed by the formula as below:
  (3) $$\rho_{r}=R+c\cdot \left(\tau_{clk}+\tau_{trop}+\tau_{iono}+\Delta t+\tau_{rev}\right)+R_{mp}+n$$
  where $\rho_{r}$ is the measurement pseudorange from receiver, Δt is the receiver clock-bias, $\tau_{rev}$ is the receiver delay, $R_{mp}$ is the multipath interference, and n is the measurement noise. Considering RF signal directly reaching the receiver via cable instead of an antenna, $R_{mp}$ can be ignored in the condition of impedance matching [21].
4. As the simulator can avoid ephemeris error, clock error, and atmosphere error, according to the Equations (2) and (3), the receiver delay can be calculated by the formula as below:
  (4) $$\tau_{rev}=\left(\rho_{r}-\rho_{s}\right)/c-\Delta t-\tau_{sim}+n$$

$\tau_{rev}$ is derived from pseudorange observation equation, which only represents the channel delay. However, Δt is calculated from positioning equation, which includes the channel delay. As the true clock-bias from pseudorange cannot be deducted in reality, we consider it is part of channel delay. In other words, under the homologous common-clock condition, receiver clock-bias is the main factor of channel delay. Thus the channel delay can be expressed by the formula as below:

(5) $$\tau_{chan}=\left(\rho_{r}-\rho_{s}\right)/c-\tau_{sim}+n$$

where $\tau_{chan}$ is the channel delay, $\rho_{s}$ is obtained from the simulator, $\rho_{r}$ is measured by the receiver, $\tau_{sim}$ is calibrated by the oscilloscope. The inevitable n can be processed by data smoothing. For PPS output delay, the measurement of time interval counter should be added into the calculation.

**Figure 4:**
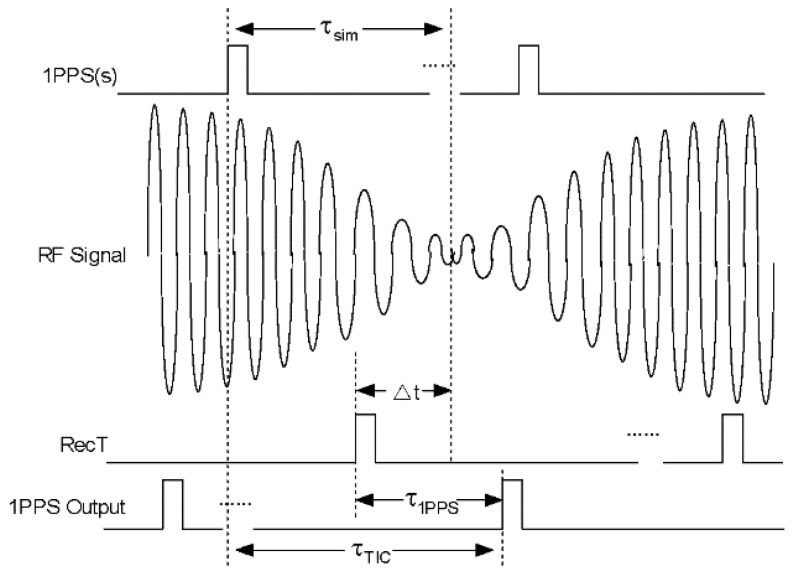
Calibration method of pulse-per-second (PPS) output delay.

5. PPS output delay mainly refers to the link delay from internal generation to hardware port, and Figure 4 is shown for a better description:

$\tau_{TIC}$ measured from time interval counter represents the bias between PPS(s) and PPS(r), and Δt calculated from pseudorange observation equation represents the bias between zero-value of RF signal and RecT. If PPS output does not exist the link delay which means $\tau_{1PPS}$ is zero, $\tau_{TIC}$ plus Δt is equal to $\tau_{sim}$. However, PPS output delay results in inequality, $\tau_{1PPS}$ can be subtracted from the formula as below:

(6) $$\tau_{1PPS}=\tau_{TIC}+\Delta t-\tau_{sim}$$

Here a precondition of RecT is leading relatively to the zero-value of RF signal. Otherwise minus should replace plus in front of Δt. Meanwhile, under different frequency band, code type, or signal modulation the receiver delay is also different, especially for the channel delay, which should be calibrated separately.

## 4. Calibration Results

### 4.1. Simulator Self-Calibration

**Figure 5:**
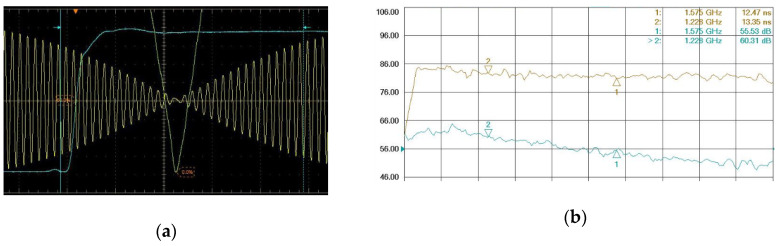
The calibration result of simulator: (**a**) time-to-code (TtC) measurement by the oscilloscope; (**b**) amplifiers delay measurement by the vector network analyzer.

The simulator (Spirent GSS8000) is set to open one channel of No.1 satellite with GEO status, and GPS L1 carrier is modulated by P-code without navigation message. As the maximum power of RF signal is about −60 dBm, two amplifiers are added behind RF signal output to ensure the oscilloscope can acquire the true signal. The oscilloscope (Tek DPO 71604C) is captured as Figure 5a,b shows the total delay of amplifiers, cables and adapters is 12.47 ns measured by a vector network analyzer(Agilent PNA-X N5242A) [10].

In order to directly find the code phase reversal point of RF signal, Hilbert transform is added into the oscilloscope. The 50% level of PPS rising edge is referred as the trigger, and the measurement of TtC is 8.24 ns.

An attenuator with delay 3.89 ns obtained from simulator product documentation should also be considered into calibration, which is added in the back RF port with low power for receiver calibration, as the above process used the front RF port with high power for simulator calibration.

(7) $$\tau_{sim}=TtC+\tau_{Attenuator}-\tau_{Amplifier}=8.24\;\mathrm{ns}+3.89\;\mathrm{ns}-12.47\;\mathrm{ns}=-0.34\;\mathrm{ns}$$

Thus, the calibration result of GPS L1 is −0.34 ns as above, and in this way, the results of other GNSS signal can be self-calibrated as well.

### 4.2. Calibration of Receiver Delay

#### 4.2.1. Channel Delay of GPS L1/L2

**Figure 6:**
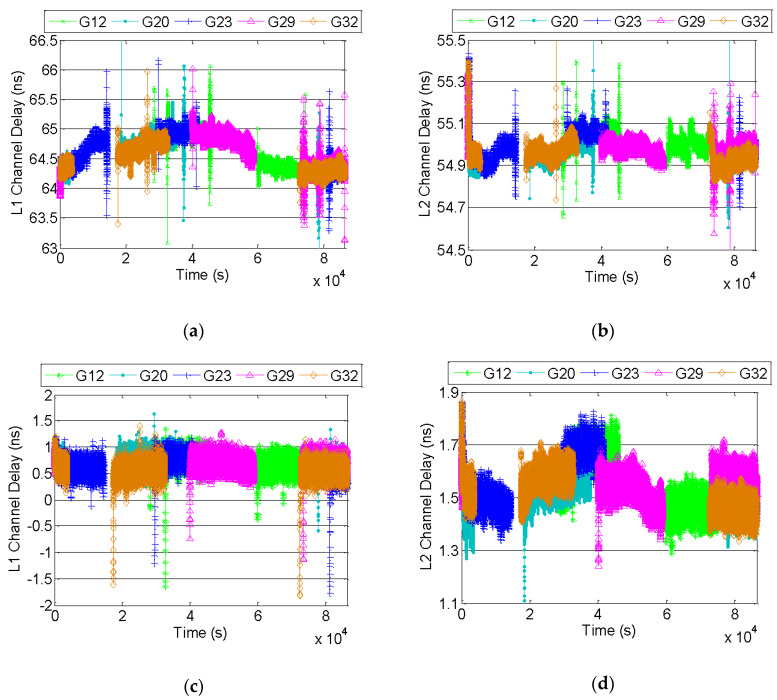
The calibration results of channel delay: (**a**) Novatel-GPS L1; (**b**) Novatel-GPS L2; (**c**) Septentrio-GPS L1; (**d**) Septentrio-GPS L2.

The 10 MHz signal is used as the external reference for the simulator and the receivers of Novatel OEMV-3G and Septentrio Polarx4 Pro. We set each receiver channel to track the specified satellite. The channel delay under GPS L1/L2 is shown as Figure 6, with the abscissa of testing time and ordinate of channel delay.

Five satellites results are selected to calculate the channel delay of each receiver. Although sometimes the channel delay fluctuated about 2 ns, the relative bias among different channels is nearly under 0.2 ns, which can be ignored in the calibration. Taking the average of channel delay as a result, the data statistics are shown in Table 1.

By contrast, the channel delay of Septentrio is under 2 ns far lower than 65 ns for Novatel, because the pseudorange from Septentrio has deducted Δt via clock-bias correction. Meanwhile, the inter-frequency bias between L1 and L2 makes a great difference, 10 ns for Novatel and 1 ns for Septentrio, which is mainly due to hardware link and filter group delay.

#### 4.2.2. Inter-Frequency Bias of GLONASS L1

From the last section, it can be seen an inter-frequency bias between GPS L1 and L2. As GLONASS uses frequency division multiple access (FDMA) technique to transmit signal, it is necessary to calibrate the inter-frequency bias among receiver channels under GLONASS L1 [2,22].

(8) $$f_{L1}^{K}[Hz]=1602.10^{6}+K\cdot 562500$$

**Figure 7:**
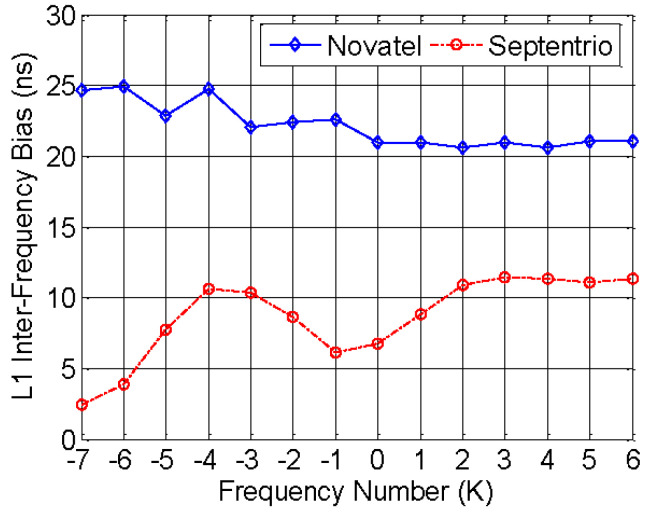
Inter-frequency bias of GLONASS L1.

GLONASS signal has 14 carrier frequencies as the frequency number K variable, and all the satellites launched after 2005 use K(−7 to 6). Ignoring the receiver channel bias under 0.2 ns, we set each channel of GLONASS L1 transmitted No.1 satellite at different K. The calibration results of two receivers are shown as Figure 7.

The results between Novatel and Septentrio differ a lot. As K from −7 to 6, the inter-frequency bias of Novatel is monotone decreasing within 5 ns, but Septentrio is monotone increasing within 10 ns. If taking −7 as the reference frequency number of GLONASS L1, the GPS/GLONASS time offset of the receiver is the inter-frequency bias at K = −7, with 24.68 ns for Novatel and 6.78 ns for Septentrio.

#### 4.2.3. PPS Output Delay

**Figure 8:**
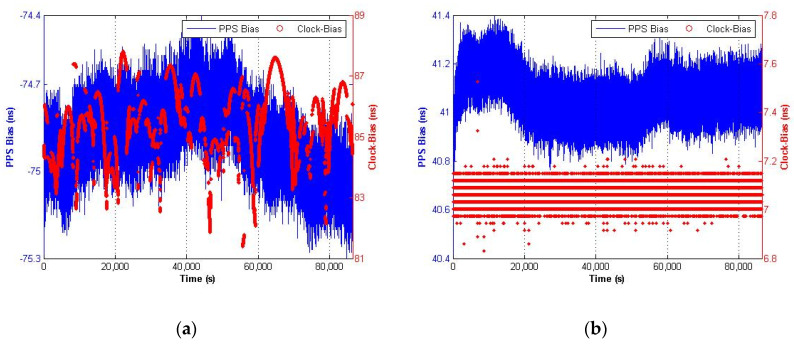
The calibration results of PPS output delay: (**a**) Novatel; (**b**) Septentrio.

After the receiver have operated stably, PPS output of the simulator and receiver are put into time interval counter of SR620, and the bias measured by SR620 is shown as Figure 8. The blue line with left ordinate represents PPS bias form SR620, and the red line with right ordinate represents clock-bias from receiver.

As PPS output of simulator is the trigger signal in SR620, SR620 PPS bias of Novatel is minus, which meant receiver time of Novatel is leading relatively to GPST. Compared with Septentrio, the clock-bias of Novatel fluctuates larger than PPS bias, thus indicates the synchronous relation between RecT and PPS output may be different, which should be analyzed with pertinence test.

Taking the average of measurement as a result, the PPS output delay statistics are shown in Table 2, with 11.05 ns for Novatel and 48.46 ns for Septentrio.

#### 4.2.4. Test on the Clock-Steering Performance of Receiver PPS Output

**Figure 9:**
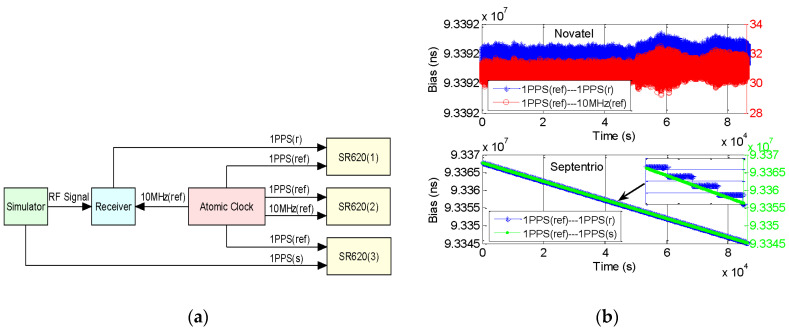
Testing of receiver PPS output: (**a**) testing method; (**b**) testing result, with the top panel of Novatel and the bottom panel of Septentrio.

Many users consider PPS output of the receiver represents GNSST. However, due to different receiver hardware, operation model, and parameter setting, PPS output may not totally synchronize with GNSST. From the last section, PPS output of Novatel is different from Septentrio. By using three sets of SR620, we design an experiment to test the clock-steering performance of receiver PPS output. The 10 MHz signal from the atomic clock of 10 MHz(ref) is only used as the external reference for the receiver, and PPS signal from the atomic clock of PPS(ref) is individually put into three sets of SR620 as the trigger signal. PPS output from the receiver of PPS(r), 10 MHz(ref) and PPS output from the simulator of PPS(s) are relatively put into SR620(1), SR620(2), and SR620(3). As the reference clock of the simulator is different from the receiver, we compare the bias of SR620(1) with SR620(2) and SR620(3) to test PPS output, with the method shown as Figure 9a.

Figure 9b shows SR620(1) bias of Novatel consistent with SR620(2), but SR620(1) bias of Septentrio consistent with SR620(3). Visibly, PPS(r) of Novatel or Septentrio is shown as the blue line steering to PPS(s). Along with the testing time PPS(r) of Septentrio synchronizes with PPS(s) shown as a green line in the bottom panel, but PPS(r) of Novatel synchronizes with 10 MHz(ref) shown as red line in the top panel. Since PPS(s) from the simulator is defined as GPST, it indicates PPS output of Novatel is maintained by the reference clock after steering to GPST, but PPS output of Septentrio is steering to GPST all the while. From the watch window in the bottom panel, PPS output of Septentrio represents the ladder-like variation of 20 ns, which reflects the synchronous precision of PPS output with 20 ns.

### 4.3. Influence of Ttp on Receiver Delay

#### 4.3.1. Relation between TtP and RecT

Both of the receivers chosen in above experiment do not have PPS input, which represents RecT from the atomic clock as the local reference. For other types of receivers, RecT will steer to the external PPS. Although the calibration method is the same as mentioned above, the calibration result especially for channel delay is affected with time-to-phase (TtP), which may be ignored by users [23].

**Figure 10:**
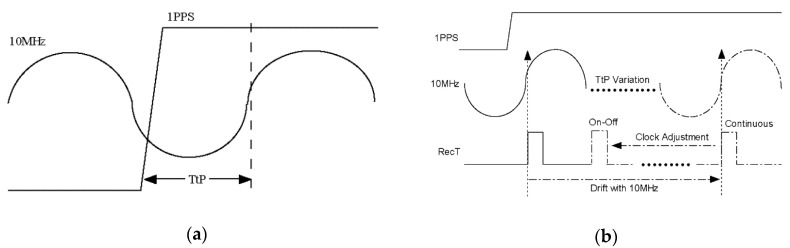
Relation between time-to-phase (TtP) and receiver time (RecT): (**a**) definition of TtP; (**b**) Ttp impact on RecT.

The phase offset between external PPS input and 10 MHz reference is named TtP, which is defined from the rising edge of PPS to zero-value of 10 MHz shown as Figure 10a [24]. For few developed receivers, internal clock system has synchronized RecT with external PPS before operating like TtP = 0, the receiver delay is not related with TtP. However, most of the commercial receivers do not manage TtP, which means RecT synchronizes with 10 MHz reference firstly and steers to PPS input secondly. Consequently, RecT will correspond to change through TtP changing, shown as Figure 10b.

While the change of 10 MHz reference results in TtP variation, it represents RecT synchronous with the change of 10 MHz phase. In the case of continuous operation, RecT is monotone changing as TtP variation, which does not adjust clock-bias after successful positioning. In the case of on–off operation, once the clock-bias exceeds adjustment threshold, the restarted receiver has to adjust clock-bias over again, which will keep RecT with the previous cycle of 10 MHz phase until clock-bias less than adjustment threshold. Thus RecT is periodic changing as TtP changing.

Whichever continuous operation or on-off operation, the change of RecT will directly affect the measurement pseudorange from the receiver, which impacts on calibration result of channel delay.

#### 4.3.2. TtP Impact on Channel Delay

**Figure 11:**
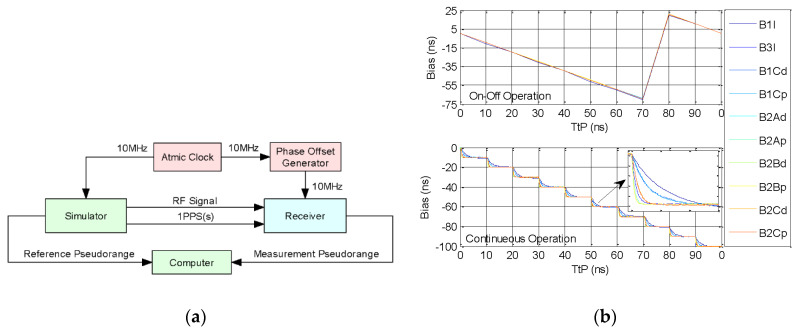
Ttp impact on channel delay: (**a**) calibration principle; (**b**) testing result, with the top panel of on–off operation and the bottom panel of continuous operation.

A representative receiver of UniNav-BDS, developed by National University of Defence Technology China, is chosen in the calibration, which is used to test the variation of channel delay through TtP changing, shown as Figure 11a [25]. The principle is almost the same as above, and the only difference is that a phase offset generator of SDI hrog-10 is added in 10 MHz reference, which is used to adjust the phase of the 10 MHz signal. Under continuous operation and on–off operation, we adjust the phase of 10 MHz reference with 10 ns every time, respectively acquiring the pseudorange bias between simulator and receiver to compare the variation of channel delay.

In the Figure 11b the abscissa represented TtP, and the ordinate represents the variation of channel delay. Visibly, under on–off operation, the channel delay represents periodic variation as TtP increased, and there is a fluctuation caused by clock adjustment when TtP is from 70 ns to 80 ns. Under continuous operation, the channel delay is monotone decreasing of 10 ns as TtP increased by 10 ns. From the watch window in the bottom panel, it can be seen the drift of channel delay for different RF signal is also different. Consequently, the receiver delay is not a fixed value, especially for the receiver having PPS input, which may change a lot along with TtP changing.

#### 4.3.3. Experiment Verification

**Figure 12:**
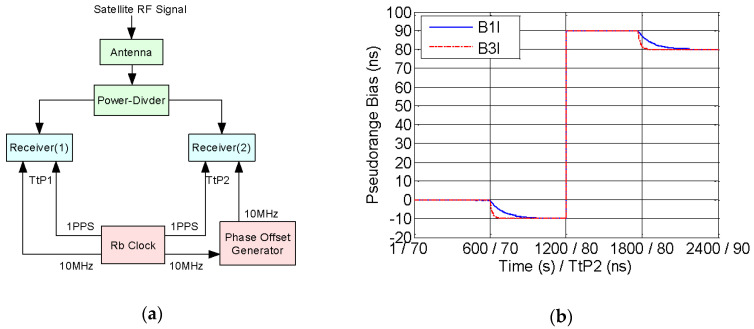
Zero-baseline comparison: (**a**) testing principle; (**b**) testing result.

In order to verify the calibration result in the last section, we use two calibrated receivers of UniNav-BDS to test a zero-baseline comparison shown as Figure 12a. The two receivers respectively track satellite RF signal from the same antenna through a power-divider, and rubidium clock of SRS FS725 provides the reference source [26,27]. The 10 MHz reference of the receiver(2) is adjusted by the phase offset generator. Maintaining TtP1 and changing TtP2, we compare the pseudorange bias between two receivers which can directly represent the variation of channel delay [28]. Satellite results of B1I and B3I are selected to verify the calibration result shown as Figure 12b. The earlier abscissa is the testing time, and the later abscissa represents TtP2 of receivers(2), and the ordinate represents pseudorange bias between two receivers.

Step 1: 0–600 s, TtP1 and TtP2 are both kept to 70 ns, and the pseudorange bias of two receivers is better than 0.5 ns.

Step 2: 600–1200 s, under continuous operation, TtP2 increases by 10 ns namely TtP2 = 80 ns. The pseudorange of the receiver(2) gradually reduces by 10 ns at first, which tends to be stable during 900–1200 s.

Step 3: 1200–1800 s, under on–off operation, both of two receivers are restarted at 1200 s, and the pseudorange of the receiver(2) jumped by 100 ns. From then on the pseudorange bias of two receivers are better than 0.5 ns like step 1.

Step 4: 1800–2400 s, under continuous operation, TtP2 increases by 10 ns namely TtP2 = 90 ns. The pseudorange of the receiver(2) also gradually reduces by 10 ns at first, which tends to be stable during 2100–2400 s like step 2.

As a result, the first step represents the calibration consistency of two receivers is better than 0.5 ns. When TtP2 increased, step 2 and step 4 respectively verify the relation between pseudorange and TtP under continuous operation, and step 3 verifies the relation under on–off operation. Therefore, the zero-baseline comparison is consistent with the calibration result between TtP and channel delay.

### 4.4. Analysis on Calibration Uncertainty

Using the simulator for absolute calibration can eliminate the influence of ephemeris error, clock error, atmosphere error, and multipath interference, which further improves the calibration precision [24,29]. There are still some calibration errors in the experiment, mainly including instrument errors and measurement errors, and it is necessary to analyze the calibration uncertainty, as shown in Table 3 [30].

The simulator are mainly reflected on TtC, like the code phase adjustment precision, RF signal stability, with uncertainty 1 ns. The oscilloscope is mainly affected by the noise ratio, with uncertainty 0.2 ns. Amplifiers delay measurement by the vector network analyzer, the amplifier, cable and adapter are mainly affected by phase noise and ripple interference, with uncertainty 0.5 ns, and the vector network analyzer is mainly reflected on group delay measurement, with uncertainty 0.2 ns. The time interval counter is mainly affected by the trigger level and channel offset, with uncertainty 0.1 ns. The pseudorange from the receiver is mainly reflected on tracking precision and measurement fluctuation, with uncertainty 0.5 ns. Receiver PPS output is mainly affected by clock stability and adjustment precision, with uncertainty 0.5 ns.

In summary, the combined uncertainty of absolute calibration can be calculated with 1.36 ns by the standard deviation.

## 5. Discussion

From the calibration results in Section 4.2, the receiver delay of different types maybe differ a lot. Meanwhile, from the calibration results in Section 4.3, the delay of the same receiver under different TtP is also different. GNSS receiver delay is not a fixed value, not only affected by temperature, SNR and aging rate, but also affected by operation model, parameter setting, reference clock, etc. It means the receiver delay may be changed in the outfield although it has just been calibrated in the laboratory, which should be paid much attention.

For the receivers without PPS input, the calibration of channel delay can also deduct clock-bias from receiver pseudorange. However, for the receivers with PPS input, although deducting the clock-bias may nearly eliminate the influence of TtP on calibration, the receiver system time will steer to GNSST instead of PPS input. Thus the receiver pseudorange cannot represent the measurement based on external PPS in the application. That is why we consider the clock-bias is part of channel delay.

Some researchers may calibrate the receiver together with the antenna in the anechoic chamber [31]. The group delay of the antenna is mainly related to frequency bands, and the inter-frequency bias of the antenna may be much larger than the receiver. In this way, the calibration result will include receiver delay, cable delay, and antenna delay. If we changes the link delay larger such as increasing the cable length, the receiver pseudorange and clock-bias will become larger synchronously. Once the clock-bias exceeds adjustment threshold, the receiver will adjust clock-bias to steer to GNSST/PPS, and the total delay may become smaller on the contrary.

## Figures and Tables

**Table 1:** Channel delay statistics under GPS L1/L2 (unit: ns).

Channel	Novatel		Septentrio	
	L1	L2	L1	L2
1	64.67	55.03	0.72	1.56
2	64.61	54.95	0.74	1.50
3	64.68	55.00	0.67	1.57
4	64.63	54.98	0.70	1.55
5	64.49	54.95	0.56	1.52

**Table 2:** PPS output delay statistics (unit: ns).

Receiver	PPS Bias	Clock-Bias	PPS Output Delay
Novatel	−74.83	85.54	11.05
Septentrio	41.07	7.06	48.46

**Table 3:** Uncertainty analysis of absolute calibration (unit: ns).

Error Source	Uncertainty
Simulator	1
Oscilloscope	0.2
Amplifier, cable, adapter	0.5
Vector network analyzer	0.2
Time interval counter	0.1
Pseudorange	0.5
Receiver PPS output	0.5
**Combined Uncertainty**	**1.36**
